# Kallmann Syndrome and MSI-H Colorectal Cancer in a Patient with a Germline *PROKR2* Variant: A Case Report with Exploratory Cohort Analysis

**DOI:** 10.3390/ijms27219703

**Published:** 2026-10-29

**Authors:** Yier Lu, Dan Li, Weiwei Liang, Ziyan Tong, Xiuyuan Zhang, Yan He, Ningjuan Yu, Shanshan Weng, Ying Yuan, Mengyuan Yang

**Affiliations:** 1 Department of Medical Oncology, Key Laboratory of Cancer Prevention and Intervention, Ministry of Education, The Second Affiliated Hospital, Zhejiang University School of Medicine, Hangzhou 310009, China; 2 Zhejiang Provincial Clinical Research Center for Cancer, Hangzhou 310009, China; 3 Department of Endocrinology, The Second Affiliated Hospital, Zhejiang University School of Medicine, Hangzhou 310009, China; 4 Cancer Center, Zhejiang University, Hangzhou 310058, China; 5 Center for Medical Research and Innovation in Digestive System Tumors, Ministry of Education, Hangzhou 310009, China

**Keywords:** colorectal cancer, Kallmann syndrome, *PROKR2*, microsatellite instability-high

## Abstract

*PROKR2* is an established gene associated with Kallmann syndrome (KS), and preclinical studies have suggested a possible role in tumor development. In this study, we report a patient with KS and microsatellite instability-high (MSI-H) colon cancer and aim to explore the potential association between *PROKR2* variants and MSI-H status in colorectal cancer (CRC). Peripheral blood samples from the proband and his parents underwent whole-exome sequencing (WES) to identify germline variants. The WES revealed that both the proband and his father carried a likely pathogenic variant in the KS-associated gene *PROKR2* (NM_144773.4:c.337T>C, p.Tyr113His). To place this case in a broader context, we performed exploratory bioinformatic analyses of somatic *PROKR2* variants in two public CRC cohorts, DFCI-CRC and TCGA-CRC, although these cohort analyses cannot inform germline risk. In the DFCI-CRC cohort, *PROKR2* somatic variant status showed no statistically significant association with MSI-H status in either the unadjusted or TMB-adjusted analysis. In the TCGA-CRC cohort, *PROKR2* somatic variants were associated with higher unadjusted odds of MSI-H (OR = 5.50, 95% CI = 1.003–30.20, *p* = 0.0248, BH-adjusted q = 0.0495). After TMB adjustment, this positive association was not retained and the adjusted estimate was in the opposite direction (adjusted OR = 0.093, 95% CI = 0.014–0.549, *p* = 0.00875, BH-adjusted q = 0.0175). Accordingly, this case report describes the co-occurrence of *PROKR2*-related KS and MSI-H CRC in a single patient, although a causal relationship between *PROKR2*-related KS and CRC remains unestablished.

## 1. Introduction

PROKR2 belongs to the G protein-coupled receptor (GPCR) family, which comprises an extracellular N-terminus and an intracellular C-terminus, as well as seven transmembrane α-helices connected by three extracellular and three intracellular loops [1]. PROKR2 shares high sequence similarity with PROKR1 and both receptors bind the same ligands, PROK1 and PROK2. They exhibit tissue-specific biological activities with different expression patterns and regulation in various human tissues [2]. Specifically, PROKR2 is highly expressed in the subventricular zone of the lateral ventricles and olfactory bulb, which serve as sources of neuronal precursors [3]. Germline variants in *PROKR2* can affect the development and migration of gonadotropin-releasing hormone (GnRH) neurons, leading to idiopathic hypogonadotropic hypogonadism (IHH) with anosmia (Kallmann syndrome, KS) or normosmic IHH (nIHH) [4]. Recent studies have expanded our understanding of the genetic and clinical spectrum associated with germline *PROKR2* variants. Martinez-Mayer and Perez-Millan reviewed 80 studies describing 435 reported cases with *PROKR2* variants. They documented 453 variant records encompassing 78 distinct variants, with KS being the most frequently reported phenotype [5].

As a G protein-coupled receptor, PROKR2 mediates intracellular signaling pathways, including calcium and cAMP signaling, upon ligand binding [2]. The prokineticin system participates in several physiological processes, including neuronal survival, pain perception, gastrointestinal smooth muscle contraction, angiogenesis, and the regulation of vascular permeability. One of the ligands of PROKR2, PROK1, also known as endocrine gland-derived vascular endothelial growth factor (EG-VEGF), promotes angiogenesis in endocrine tissues [6]. Together, these observations have prompted further investigation into the potential role of *PROKR2* in tumorigenesis. In colorectal cancer (CRC), Goi et al. [7] reported that positive immunohistochemical staining for PROKR2 was associated with a greater risk of vascular invasion, lymph node metastasis, liver metastasis, and hematogenous metastasis in patients with CRC, suggesting its potential value as a prognostic marker in CRC. Additionally, Garcia-Etxebarria et al. [8], based on multi-omics analyses of CRC and adenoma, reported that the germline *PROKR2* SNP rs6085078 was associated with high CRC risk in their cohort. However, the potential relationship between the endocrine disorder KS, caused by *PROKR2* variants, and the development of CRC remains unclear. Here, we report a Chinese patient with KS and microsatellite instability-high (MSI-H) colon cancer who carried a likely pathogenic germline variant in *PROKR2*. Motivated by this case, we conducted exploratory analyses of public CRC cohorts to examine the associations of somatic *PROKR2* variants with MSI-H status and clinical outcomes.

## 2. Results

### 2.1. Case Presentation and Family Genetic Mutation Analysis

The proband was a 26-year-old Chinese man who initially presented at a local hospital in 2023 with recurrent abdominal pain and diarrhea. Gastrointestinal endoscopy revealed chronic atrophic gastritis and a large mass in the ascending colon. On 15 June 2023, he underwent laparoscopic partial colectomy with lymph node dissection. Postoperative histopathology revealed moderately to poorly differentiated adenocarcinoma with a mucinous component and invasion into the subserosa. Cancer metastasis was detected in 1/58 regional lymph nodes. Preoperative chest and abdominal CT scans did not reveal distant metastasis, suggesting that the pathological stage was pT3N1aM0. Immunohistochemistry showed loss of MSH2 and MSH6 expression in the tumor and retained MLH1 and PMS2 expression. Molecular testing identified a *KRAS* G13D mutation and a *TP53* frameshift variant (p.Arg47fsTer76), and confirmed MSI-H status; no somatic variants were detected in *MSH2* or *MSH6*, and TMB data were unavailable. The patient had a history of developmental delay and olfactory dysfunction and had been clinically diagnosed with KS, agenesis of the olfactory bulbs and tracts, at the age of 18. He was previously treated with 2000 U of human chorionic gonadotropin (HCG) weekly via intramuscular injection but that had been discontinued because of increasing body weight. He noted slight enlargement of his testicles and sparse pubic hair growth. A hormone level assessment on 11 July 2023 revealed decreased levels of luteinizing hormone (LH) at 0.24 IU/L (normal range: 1.50–9.30 IU/L), a progesterone concentration lower than 0.67 nmol/L (normal range: 0.89–3.88 nmol/L), a testosterone concentration of 0.32 nmol/L (normal range: 4.27–28.24 nmol/L), and a calculated free Androgen index of 1.07 (normal range: 11.14–81.01). His FSH level was within the normal range (1.88; normal range: 1.40–18.10 IU/L), and no clinically significant abnormalities were observed in the levels of the other hormones tested (Supplementary Table S1). On 11 July 2023, the patient began adjuvant chemotherapy with one cycle of XELOX, consisting of intravenous oxaliplatin (130 mg/m^2^) on day 1 and oral capecitabine (1000 mg/m^2^ twice daily) on days 1–14. Following the first cycle, hepatic dysfunction prompted a switch to mFOLFOX6. He received 11 cycles from 3 August to 29 December 2023. Each cycle consisted of intravenous oxaliplatin (85 mg/m^2^) and leucovorin (400 mg/m^2^) on day 1, followed by an intravenous bolus of fluorouracil (400 mg/m^2^) and a continuous intravenous infusion of fluorouracil (2400 mg/m^2^) over 46 h. Follow-up assessments through October 2026 showed no evidence of recurrence or metastasis.

**Figure 1:**
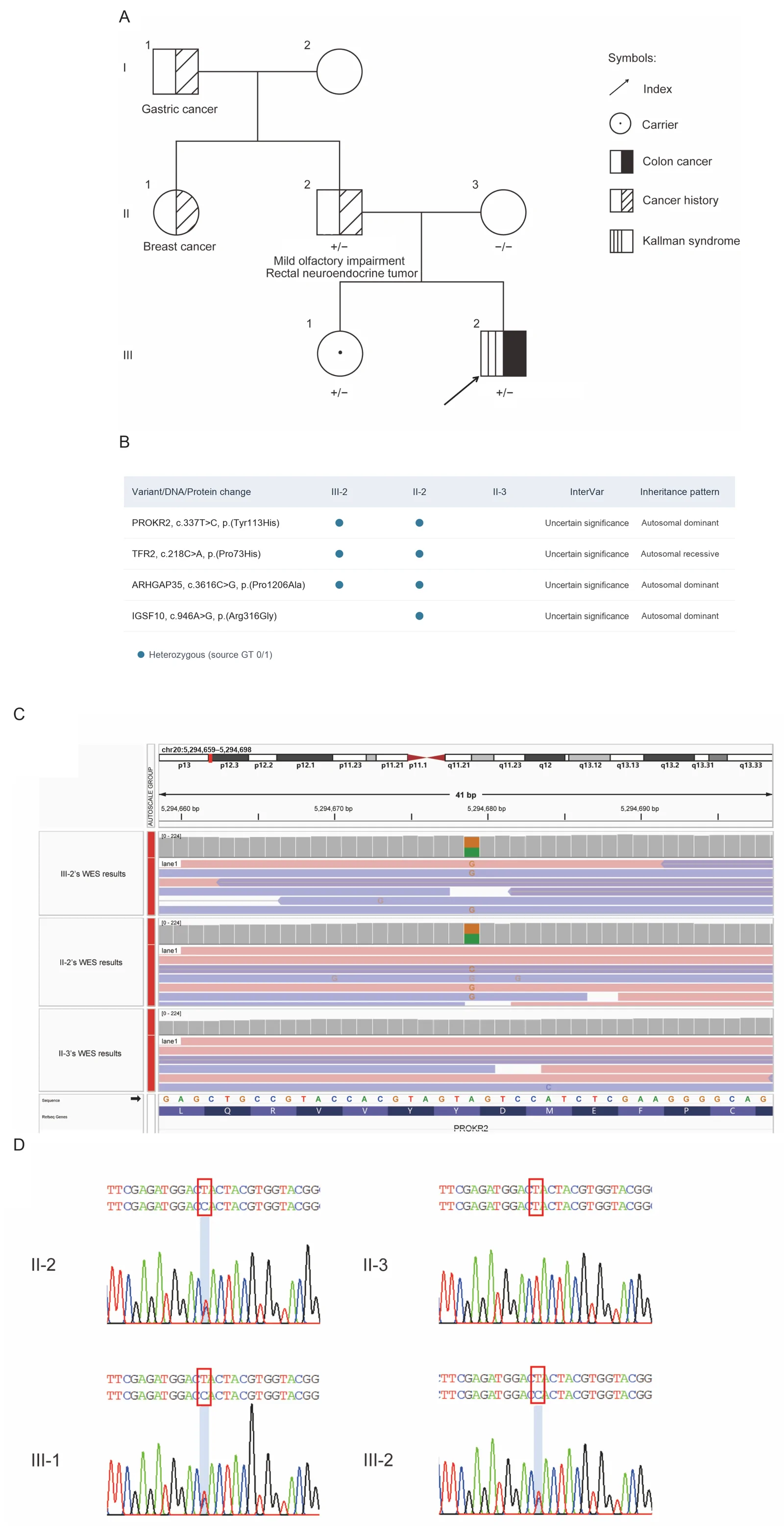
Pedigree and genetic findings in the proband and relatives. (**A**) Pedigree of this family carrying the *PROKR2* NM_144773.4:c.337T>C, p.Tyr113His variant and cancer history. (**B**) All variants initially classified as variants of uncertain significance in the 30 hereditary cancer predisposition genes and 58 CHH-associated genes evaluated by WES. (**C**) Variant status of the *PROKR2* NM_144773.4:c.337T>C, p.Tyr113His site. The red triangle marks the centromeric constriction, a highly repetitive region where read mapping may be unreliable. The red square indicates the interval shown in the enlarged view (chr20:5,294,659–5,294,698). (**D**) Sanger sequencing of *PROKR2* variants. Red boxes indicate the c.337T>C site. +/− represents the heterozygous variant, and −/− represents the wild type.

As for the family history, his father had mild olfactory impairment and a history of a rectal neuroendocrine tumor, his aunt had a history of breast cancer, and his grandfather had a history of gastric cancer (Figure 1A). Given the proband’s KS, early-onset MSI-H colon cancer, and family history of malignancy, we performed WES on the proband and his parents to investigate potential germline causes of his clinical presentation. The analysis included 30 genes associated with hereditary cancer predisposition (Supplementary Table S2) and 58 genes associated with congenital hypogonadotropic hypogonadism (CHH) (Supplementary Table S3). Initial interpretation of the WES data did not identify any pathogenic or likely pathogenic variants in the genes evaluated. All variants initially classified as variants of uncertain significance (VUS) are summarized in Figure 1B. WES-based copy number variation (CNV) calling showed no apparent abnormality or large deletion in *MSH2*, *MSH6* and *EPCAM*. A molecular diagnosis of LS was therefore not established. Further evaluation of the CHH-associated variants, considering the mode of inheritance and the proband’s clinical phenotype, prioritized two heterozygous missense variants for reassessment: *PROKR2* (NM_144773.4:c.337T>C; p.Tyr113His) and *ARHGAP35* (NM_004491.4:c.3616C>G; p.Pro1206Ala). Both variants were identified in the proband and his father but were not detected in his mother. Sanger sequencing confirmed the familial distribution of the *PROKR2* variant and additionally identified it in the proband’s elder sister (Figure 1D). Reassessment under the ACMG/AMP framework, incorporating the familial segregation findings and available published evidence, led to reclassification of the *PROKR2* variant as likely pathogenic. The *ARHGAP35* variant remained a VUS because the available evidence was insufficient for upgrading its classification (Table 1). Together with the proband’s clinical phenotype, these findings supported a diagnosis of *PROKR2*-related KS.

### 2.2. Exploratory Analyses of Tumor-Reported PROKR2 Variants in Public CRC Cohorts

**Figure 2:**
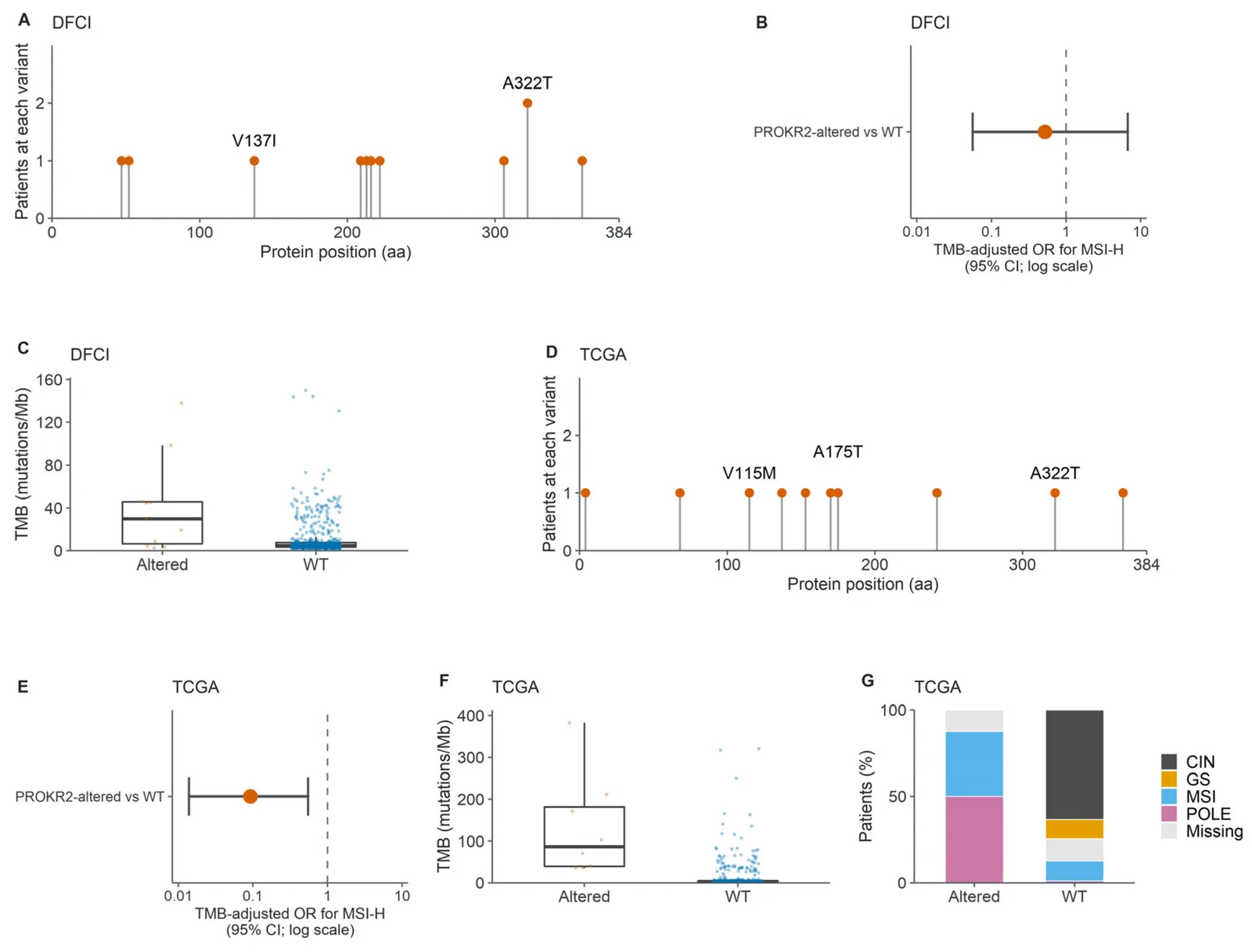
Bioinformatics analysis of the DFCI and TCGA databases. (**A**) Variant status of the *PROKR2* gene in the DFCI-CRC database. (**B**) Comparison of TMB-adjusted odds of MSI-H status between the *PROKR2*-altered group and the WT group in the DFCI-CRC cohort using Firth logistic regression. (**C**) Comparison of TMB levels between the *PROKR2*-altered group and the WT group in the DFCI-CRC database using a two-sided Wilcoxon rank-sum test. (**D**) Variant status of the *PROKR2* gene in the TCGA-CRC database. (**E**) Comparison of TMB-adjusted odds of MSI-H status between the *PROKR2*-altered group and the WT group in the TCGA-CRC cohort using Firth logistic regression. (**F**) Comparison of TMB levels between the *PROKR2*-altered group and the WT group in the TCGA-CRC database using a two-sided Wilcoxon rank-sum test. (**G**) Comparison of tumor subtypes between the *PROKR2*-altered group and the WT group in the TCGA-CRC database using a Fisher-Freeman-Halton exact test with Monte Carlo estimation.

To further investigate the correlation between *PROKR2* variants and MSI-H status, as well as the potential prognostic relevance of *PROKR2* variants in CRC, we conducted bioinformatics analyses on data from public databases. Notably, the germline *PROKR2* p.Tyr113His variant identified in this family was not observed as a somatic variant in the analyzed public CRC cohorts. Analysis of the DFCI-CRC database revealed that among 619 colorectal cancer patients, 11 harbored *PROKR2* variants (Figure 2A). In the DFCI-CRC cohort, MSI status was based on the categorical DFCI clinical variable, recorded as “MSI-high” or “MSS”. Among the 529 patients with available MSI status data, those harboring somatic *PROKR2* variants (*n* = 9) had higher odds of MSI-H than *PROKR2* wild-type (WT) cases (*n* = 520) (OR = 3.97, 95%CI 0.77–18.83, *p* = 0.0517, BH-adjusted q = 0.0517). However, this association was not retained after adjustment for TMB (adjusted OR = 0.52, 95% CI = 0.056–6.71; *p* = 0.592; BH-adjusted q = 0.592). (Figure 2B). Additionally, TMB rank distributions differed between patients with and without *PROKR2* alterations (two-sided Wilcoxon rank-sum *p* = 0.0060) (Figure 2C). Although the *PROKR2*-altered group had a higher observed median TMB (median difference was 24.98 mutations/Mb), the 95% bootstrap CI (−0.63–41.67) included zero, indicating that this median-difference estimate was imprecise.

**Figure 3:**
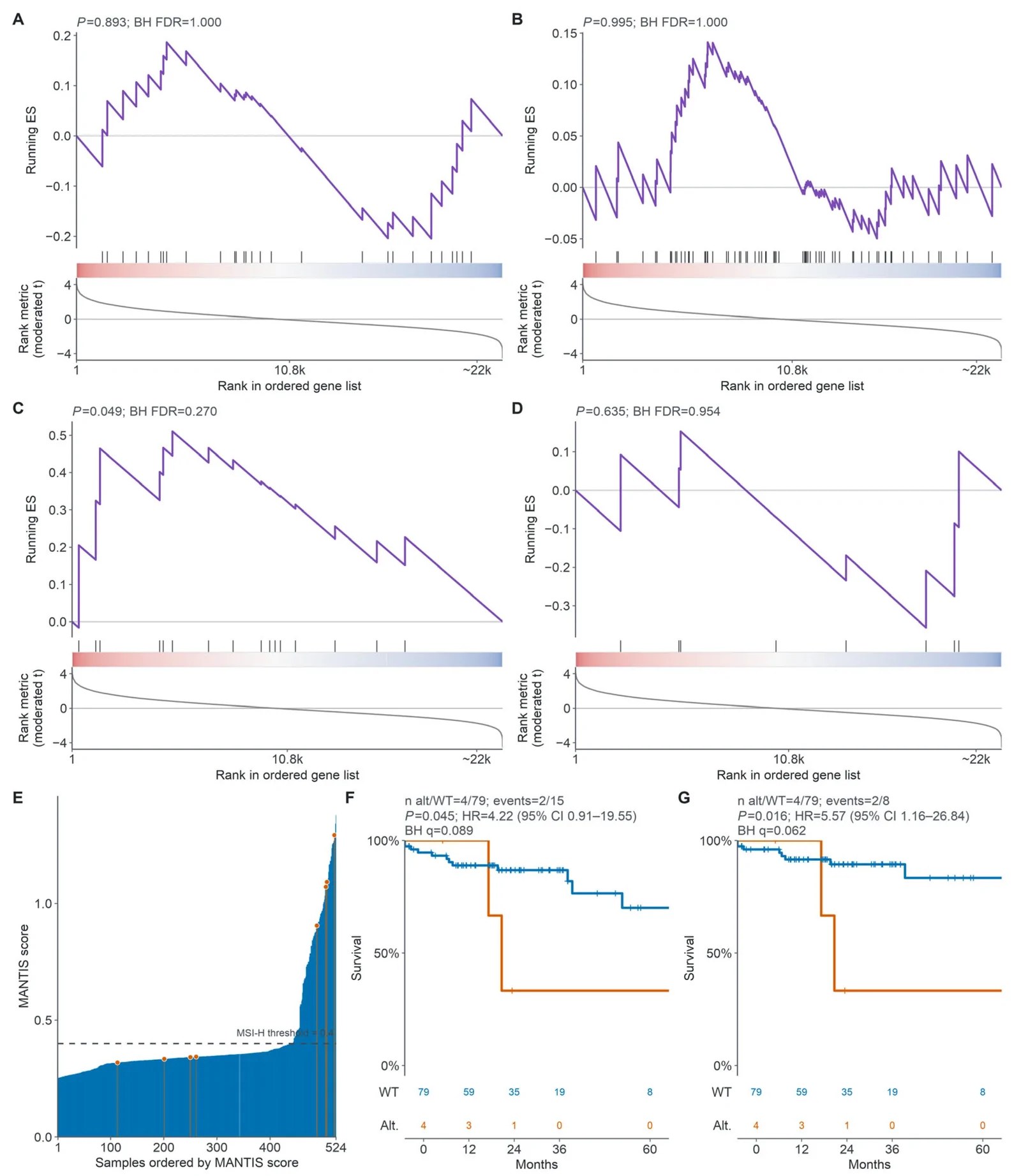
Functional and survival analysis of the TCGA database. (**A**–**D**) Gene set enrichment analysis of genes ranked by the MC3-defined *PROKR2* alteration jointly adjusted for MSI status, log_10_ TMB, and *POLE* alteration status in TCGA-CRC tumors. The color strip represents the moderated t statistic for *PROKR2*-altered versus WT tumors, with red indicating positive values, blue indicating negative values, and white indicating values near zero. (**E**) Ranked bar plot of MSI-MANTIS scores for individual tumors in the TCGA-CRC cohort. Each bar represents one tumor sample, with samples ordered by MSI-MANTIS score. Tumors were classified as MSI-H or MSS using a cutoff of 0.4. Survival analysis of OS probability (**F**) and DSS probability (**G**) between patients with *PROKR2* variants and WT patients among those MSI-H patients. *p* values are from log-rank tests; HRs and CIs are from univariable Cox models.

Analysis of the TCGA-CRC database revealed that among 526 CRC patients, eight had a total of 10 *PROKR2* variants; of these eight patients, seven harbored one variant each and one harbored three distinct *PROKR2* variants (Figure 2D). In the TCGA-CRC cohort, patients with *PROKR2* somatic variants were more likely to exhibit MSI-H status (OR = 5.50, 95%CI 1.003–30.20, *p* = 0.0248, BH-adjusted q = 0.0495). However, after adjustment for TMB, *PROKR2* variant status was significantly related to MSI-H status but showed an effect in the opposite direction (adjusted OR = 0.093, 95% CI = 0.014–0.549; *p* = 0.00875; BH-adjusted q = 0.0175) (Figure 2E). Moreover, patients with *PROKR2* variants had significantly higher TMB than did those without *PROKR2* gene variants (median difference was 83.38 mutations/Mb, 95% bootstrap CI 33.00–207.83, *p* = 8.29 × 10^−6^) (Figure 2F). In addition, tumor subtype distributions differed significantly between the two groups. Specifically, the *PROKR2*-variant group had a higher proportion of MSI and *POLE*-mutant CRC subtypes than the group without *PROKR2* variants (Cramér’s *V* = 0.487, *p* = 5 × 10^−6^) (Figure 2G). The subtype, MANTIS score, and TMB for each of the eight tumors are provided in Supplementary Table S4. Considering that the higher TMB in *PROKR2*-variant tumors may be attributable to passenger mutations arising in hypermutated tumors, we compared *PROKR2* with 20 other genes of similar coding length and matched WT expression levels. Among the matched control genes, TMB differences in mutation carriers for 11 genes in DFCI and 16 genes in TCGA passed global BH correction (Supplementary Table S5). The results showed that this difference was not unique to *PROKR2*. Patients in the TCGA-CRC cohort were stratified by *PROKR2* variant status into *PROKR2*-altered and WT groups for differential gene expression analysis and gene set enrichment analysis (GSEA). GSEA showed that the Watanabe and Koinuma MSI-up signatures were enriched towards the *PROKR2*-altered end of the ranked list (NES = 2.27 and 2.55, respectively). The corresponding MSI-down signatures showed negative NES values and were enriched toward the WT end (NES = −2.78 and −1.91). All four signatures remained significant after BH correction (FDR = 2.7 × 10^−4^, 2.2 × 10^−12^, 1.2 × 10^−6^, and 0.003, respectively). However, after joint adjustment for MANTIS-defined MSI status, log_10_ TMB, and *POLE* alteration status (*n* = 522; 8 *PROKR2*-altered and 514 WT tumors), none of the four MSI signatures remained significant after BH correction (all FDR ≥ 0.270; Figure 3A–D). NES, nominal *p* values and BH-adjusted FDR values are provided in Supplementary Table S6.

Tumors from the TCGA-CRC database were classified as MSI-H or MSS using MANTIS scores retrieved from the cBio-Cancer Genomics Portal (cBioPortal), with the recommended cutoff of 0.4 (Figure 3E). Within the MSI-H group, further survival analysis was conducted based on the *PROKR2* variant status (4 *PROKR2*-altered and 79 WT). The results indicated that, in the MSI-H subgroup, patients without *PROKR2* variants had longer overall survival (OS) (HR = 4.22, 95%CI 0.91–19.55, *p* = 0.045, BH-adjusted q = 0.089) and disease-specific survival (DSS) (HR = 5.57, 95%CI 1.16–26.84, *p* = 0.016, BH-adjusted q = 0.062) than those with *PROKR2* variants (Figure 3F,G). Although nominal *p* values for OS and DSS were below 0.05, neither association remained significant after BH correction. Given the small variant group (*n* = 4), these results should be interpreted cautiously. Progression-free interval (PFI) did not differ significantly between the *PROKR2*-altered and WT groups (*p* = 0.117). An informative comparison of disease-free interval (DFI) was not possible because only one variant carrier was included in the DFI analysis, and this patient experienced no events during follow-up (Supplementary Figure S1A,B). Firth-penalized Cox sensitivity analyses for sparse data yielded imprecise estimates. None of the associations were significant after BH correction across the four prespecified endpoints (q = 0.111, 0.111, and 0.134 for OS, DSS, and PFI, respectively) (Supplementary Table S7).

## 3. Discussion

In this study, we report a male patient carrying a likely pathogenic germline variant in *PROKR2* who was diagnosed with KS at age 18 and MSI-H colon cancer at age 26. Motivated by this case, we conducted exploratory bioinformatics analyses in two public CRC cohorts, DFCI-CRC and TCGA-CRC. In the TCGA-CRC cohort, although unadjusted analyses showed a higher observed MSI-H proportion in *PROKR2*-altered tumors, the direction of the association reversed after adjustment for TMB. After adjustment for MANTIS-defined MSI status, log_10_TMB, and *POLE* alteration status, neither the Watanabe nor Koinuma MSI signature remained enriched after BH correction. Together, these findings limit support for a *PROKR2*-specific MSI-related signal.

MSI results from Mismatch repair (MMR) deficiency, which allows DNA replication errors to persist and leads to changes in the length of microsatellite sequences [13]. Approximately 15% of CRCs exhibit MSI-H, which is attributed to (i) epigenetic silencing of *MLH1*, (ii) germline mutations in one of the mismatch repair genes (*MLH1*, *MSH2*, *MSH6*, or *PMS2*) or *EPCAM* deletion, or (iii) biallelic somatic MMR mutations [14]. Although WES did not identify any pathogenic or likely pathogenic germline variants in the MMR genes, it may miss certain copy-number changes, complex structural rearrangements, and deep intronic or regulatory variants. Further genetic evaluation should therefore be considered, including multiplex ligation-dependent probe amplification (MLPA) to assess deletions or duplications involving *MSH2* and *EPCAM*, and long-read genome sequencing to investigate complex structural and noncoding variants. Taken together, the tumor’s MSI-H phenotype is most consistent with an MMR alteration that was not detected by WES. Given the patient’s early-onset CRC, family history of cancer, and unexplained MSH2/MSH6 loss, the findings remain suggestive of a Lynch-like syndrome. Close clinical follow-up and colonoscopic surveillance are warranted while further molecular evaluation is pursued.

*PROKR2* is an established KS-associated gene, with human genetic studies implicating impaired prokineticin signaling in abnormalities of olfactory and reproductive development [15]. Evidence relevant to the p.Tyr113His variant includes both clinical observations and functional studies. Cole et al. identified this variant in a heterozygous female patient with KS and demonstrated reduced receptor expression and impaired downstream signaling, including intracellular calcium mobilization [9]. Ha et al. subsequently described a male patient with KS carrying the same heterozygous variant [11]. These findings provide support for the contribution of p.Tyr113His to the proband’s phenotype.

However, the clinical manifestations of heterozygous *PROKR2* variants are variable. Clinically unaffected carriers of p.Tyr113His have been reported, suggesting incomplete penetrance [11,12]. In the present family, the proband had typical KS, whereas his father reported mild hyposmia and his carrier sister had no typical KS phenotype. These differences highlight the difficulty of predicting clinical manifestations in heterozygous carriers and the importance of interpreting genetic findings in the context of each individual’s phenotype.

Other variants identified in this family were evaluated in light of the evidence for their respective gene–disease associations. Lippincott et al. reported heterozygous *ARHGAP35* variants in patients with KS or normosmic IHH and provided supporting evidence from zebrafish models and functional assays [16]. Nevertheless, their findings particularly implicated protein-truncating variants and selected missense variants within the RhoGAP domain. No variant-specific functional evidence was identified for p.Pro1206Ala, which therefore remained a VUS. *TFR2*-related hemochromatosis is an autosomal recessive disorder that can cause secondary hypogonadotropic hypogonadism through iron overload, and a single heterozygous variant is insufficient to establish this diagnosis [17]. Accordingly, the heterozygous *TFR2* p.Pro73His variant did not provide a sufficient genetic explanation for the proband’s phenotype.

Oligogenic inheritance has been reported in KS and other forms of congenital GnRH deficiency, including cases involving heterozygous *PROKR2* variants [18]. This provides a rationale for considering the co-occurring *ARHGAP35* and *TFR2* variants in this family. However, the *ARHGAP35* p.Pro1206Ala variant remains a VUS, and the single heterozygous *TFR2* variant does not establish a diagnosis of the associated recessive disorder. Their co-occurrence therefore does not establish an oligogenic explanation for the proband’s phenotype, and evidence of their individual effects and combined functional consequences would be required. Overall, the proband’s characteristic phenotype and the published clinical and functional evidence favor *PROKR2* p.Tyr113His as the most plausible genetic explanation for his KS.

Whether KS is associated with an increased or decreased risk of CRC remains unclear, as direct clinical evidence is limited. Eidbo and Barnett reported cancer outcomes among women aged ≥50 years with and without KS in a 2026 ASCO conference abstract. Among those not receiving hormone replacement therapy (HRT), the matched cohort without KS was reported to have a higher risk of CRC than the cohort with KS [19]. Given the unexplained internal inconsistencies in the sample sizes and statistical results reported in this abstract, we interpret its conclusions cautiously. Moreover, this preliminary finding was restricted to women aged ≥50 years and does not establish the relationship between KS and CRC risk in the broader KS population.

Given the limited evidence specific to KS, experimental and epidemiological studies of hormonal exposures provide indirect context for exploring a possible relationship. Ovariectomy increased tumor formation and the proportion of MSI-positive tumors in a rat model, while former HRT use was associated with an increased risk of MSI-positive colon cancer in women [20,21]. Because the proband is male, observational studies have reported an increased risk of CRC among men with prostate cancer who underwent orchiectomy or received androgen-deprivation therapy (ADT) [22,23]. However, neither study assessed tumor MSI status. Thus, these data provide only indirect evidence and do not establish an association between KS or male hypogonadism and MSI-H CRC.

In the present case, the proband with *PROKR2*-related KS had received human chorionic gonadotropin therapy, which was discontinued because of adverse effects. Subsequent testing showed low LH and testosterone levels, consistent with persistent hypogonadism. This observation raises the possibility that KS-associated sex hormone deficiency could be relevant to his colorectal cancer. However, the coexistence of KS and MSI-H colon cancer in a single patient does not establish a causal relationship. Further studies are needed to determine whether KS-associated hypogonadism modifies CRC risk and whether any such association differs according to tumor MSI status. Alternative explanations for the coexistence of KS and MSI-H CRC should also be considered. The patient’s tumor may have arisen through an MMR defect unrelated to KS, or the two conditions may have occurred together by chance. A possible contribution of local PROK1 (EG-VEGF)–PROKR2 signaling within tumor tissue also warrants consideration. The available data do not distinguish among these possibilities.

The proband was diagnosed with stage III deficient MMR (dMMR) colon cancer in 2023, underwent curative resection, and subsequently received adjuvant fluoropyrimidine-oxaliplatin doublet chemotherapy. Results from the ATOMIC trial, presented at the 2025 ASCO Annual Meeting, showed that adding atezolizumab to adjuvant mFOLFOX6 improved disease-free survival compared with mFOLFOX6 alone [24]. Accordingly, in 2025, the NCCN Guidelines first listed FOLFOX or CAPEOX plus atezolizumab as preferred adjuvant treatment options for patients with resected stage III dMMR/MSI-H colon cancer [25]. Therefore, it raises the question of what should be considered when administering immunotherapy to patients with KS. The European Society of Endocrinology guideline states that a controlled endocrinopathy is not a contraindication to initiating or continuing immune checkpoint inhibitor (ICI) therapy [26]. If ICI therapy were considered for this patient, management should involve collaboration between oncology and endocrinology. Baseline assessment should document his gonadal dysfunction and evaluate adrenal and thyroid function. Regular monitoring would help distinguish new endocrine toxicities from his pre-existing condition and guide individualized hormone replacement decisions [26].

Our research also has several limitations. Firstly, the sample size for some of the results is relatively small, which may limit the generalizability and representativeness of the findings. Secondly, the proposed association between germline *PROKR2* variants and susceptibility to MSI-H CRC has not been experimentally validated. Ideally, CRC risk should be assessed in a large cohort of individuals with KS and/or germline *PROKR2* variants and compared with that in the general population. Because such cohort data were not accessible, we instead conducted an exploratory analysis of somatic *PROKR2* variants in publicly available cancer datasets. This analysis does not directly assess CRC risk associated with KS or germline *PROKR2* variants. Thirdly, hypermutation and MSI status remain potential confounders. Because *PROKR2*-altered tumors were more frequently MSI-H, differences in TMB and enrichment of MSI-related gene sets may partly reflect MSI status rather than a *PROKR2*-specific association. Lastly, the absence of tumor PROK1/PROKR2 expression and downstream-signaling analyses, together with the lack of tumor *PROKR2* sequencing and loss-of-heterozygosity assessment, limited our ability to evaluate local pathway activity or whether the germline *PROKR2* allele had acquired a second somatic hit.

In summary, we report a patient with *PROKR2*-related KS carrying a likely pathogenic germline variant (NM_144773.4: c.337T>C, p.Tyr113His) who developed MSI-H colon cancer. Exploratory analyses of public CRC cohorts suggested an association between tumor *PROKR2* variants and MSI-H status, with poorer survival observed among variant carriers in a small MSI-H subgroup. These findings are hypothesis-generating and do not establish that *PROKR2*-related KS or associated hypogonadism increases CRC risk or contributes to MSI-H status. Validation in larger cohorts and mechanistic studies is needed to clarify these relationships.

## 4. Materials and Methods

### 4.1. Patients and Genomic DNA Samples

This study was approved by the Ethics Committee of the Second Affiliated Hospital, Zhejiang University School of Medicine (Approval No. 2024.0888), and informed consent was obtained from the family pedigree. The study adhered to the ethical principles of the Declaration of Helsinki and Good Clinical Practice guidelines. Genomic DNA samples were extracted from the peripheral blood of patients via a QIAamp DNA Blood Midi Kit (Qiagen, Hilden, Germany).

### 4.2. Whole-Exome Sequencing and Variant Analysis

Whole-exome sequencing, which can also be used to detect CNVs, was conducted by iGeneTech (Beijing, China). The exonic regions of the genomic DNA were captured via the Agilent SureSelect Human All Exon V7, with the hg19 human reference genome used as a reference. Sequencing reads were analyzed, and variants were further detected via the GATK4 tool based on the Linux system. Mutations were subsequently annotated via the ANNOVAR tool (version 201707). The protein mutation status of the *PROKR2* p.Tyr113His site in the family was visualized via IGV software (version 2.19.8) [27]. The obtained annotation files were further processed via the maftools tool in R. The “annovarToMaf” function within maftools was utilized to convert the integrated file of mutations into a mutation maf file. Subsequently, visual analysis of *PROKR2* was performed via “lollipopPlot”. The WES data passed quality control, and all samples met the required quality thresholds.

### 4.3. Sanger Sequencing

The variant c.337T>C of the *PROKR2* gene (NM_144773.4) was detected with Sanger sequencing using the following specific primers: *PROKR2*-F (5′-TGAGGACATGACCAAGACCC-3′) and *PROKR2*-R (5′-ATCCTCACCTGTCAATGGCA-3′). The results were visualized with the “sangerseqR” R package.

### 4.4. Cohort Features and Statistical Comparisons

We selected two colorectal cancer cohorts, “Colorectal Adenocarcinoma (DFCI, Cell Reports 2016)” (DFCI-CRC) and “Colorectal Adenocarcinoma (TCGA, PanCancer Atlas)” (TCGA-CRC), to assess the associations of somatic *PROKR2* alteration status with MSI status, TMB, and molecular subtype. In DFCI-CRC, somatic mutation data were derived from comparisons of WES data between matched tumor and normal samples. MSI status was obtained from the cBioPortal clinical annotation and classified as MSI-H or MSS. In TCGA-CRC, MSI status was assigned using the cBioPortal MSI-MANTIS score, with MSI-H defined as a score ≥ 0.4. Unadjusted MSI-H versus MSS distributions were compared using two-sided Fisher’s exact tests; odds ratios (ORs) and 95% confidence intervals (CIs) were calculated. Plotted MSI-H proportions are shown with exact binomial 95% CIs. TMB distributions were compared using two-sided Wilcoxon rank-sum tests with asymptotic *p* values, tie correction, and continuity correction. Median differences and 95% CIs were estimated using a stratified percentile bootstrap with 5000 resamples. As an exploratory sensitivity analysis, we fitted Firth logistic regression models separately in each cohort, with MSI-H status as the outcome and *PROKR2* alteration status plus z-scored log_2_(1 + source-provided TMB [mutations/Mb]) as predictors. These models included samples with nonmissing MSI status and TMB; conditional ORs and profile-likelihood 95% CIs were reported, and likelihood-ratio *p* values were calculated. BH adjusted q values were calculated separately for the two cohort-specific unadjusted MSI-status comparisons, the three TMB/molecular-subtype tests, and the two prespecified cohort-specific TMB-adjusted models.

### 4.5. RNA Sequencing and GSEA

Raw count-format RNA-sequencing data for TCGA colon adenocarcinoma (TCGA-COAD) and rectal adenocarcinoma (TCGA-READ) were queried and downloaded from the Genomic Data Commons (GDC) using the “TCGAbiolinks” workflow, beginning with “GDCquery”. Expression profiles from TCGA-COAD and TCGA-READ were merged by Ensembl gene identifier to generate a combined TCGA colorectal cancer (TCGA-CRC) dataset. Normal-tissue samples were excluded according to the sample-type component of TCGA barcodes. One primary-tumor RNA-seq sample was retained per patient before matching expression data to patient-level mutation status.

GDC VarScan2 somatic mutation MAF files were separately obtained for TCGA-COAD and TCGA-READ using the WXS experimental strategy, the “Simple Nucleotide Variation” data category, the “Annotated Somatic Mutation” data type, and the “VarScan2 Annotation” workflow. These MAF files were used only for secondary caller-level comparison and did not define mutation status. Somatic mutation status was defined exclusively using the cBioPortal PanCancer Atlas MC3 callset. Patients carrying at least one non-synonymous coding or splice-site *PROKR2* variant were classified as *PROKR2*-altered; synonymous and non-coding variants were excluded. RNA-seq counts were used only for expression quantification and were matched to MC3-defined mutation status using TCGA barcodes.

After barcode matching, 524 TCGA-CRC tumors were included in the expression analysis, comprising 8 *PROKR2*-altered and 516 WT tumors. Lowly expressed genes were filtered using edgeR’s “filterByExpr”, and library sizes were normalized using the trimmed mean of M-values method implemented in edgeR’s “calcNormFactors”. For the primary Figure 3A–D analysis, differential expression was assessed using limma-voom with empirical Bayes moderation in a joint model containing MC3-defined *PROKR2* status, MANTIS-defined MSI status, log_10_-transformed TMB (nonsynonymous mutations/Mb), and MC3-defined *POLE* alteration status. Because MANTIS-defined MSI status was a model covariate, two WT tumors without MANTIS scores were excluded, leaving 522 tumors (8 *PROKR2*-altered and 514 WT tumors) for model fitting. Genes were ranked by the moderated t statistic for the *PROKR2*-altered coefficient, with positive values indicating higher expression in *PROKR2*-altered tumors after adjustment for MSI status, TMB, and *POLE* alteration status. Ensembl stable IDs were mapped to Entrez IDs using “org.Hs.eg.db” (v3.14.0). For multiple Ensembl features mapping to the same Entrez ID, the feature with the highest average expression was retained; ties were resolved by Ensembl ID.

Preranked gene set enrichment analysis was performed using “fgseaMultilevel” with the MSigDB human C2 collection, including C2:CP and C2:CGP gene sets (release 2026.1.Hs; Entrez-based GMT). The moderated t statistic for the covariate-adjusted *PROKR2* coefficient was used as the ranking metric. The four displayed signatures were Watanabe MSI-up, Watanabe MSI-down, Koinuma MSI-up, and Koinuma MSI-down. Gene sets with 5–500 genes overlapping the ranked list were eligible for testing. Nominal enrichment *p* values, normalized enrichment scores (NES), and leading-edge genes were calculated for each eligible gene set. The leading edge comprised genes contributing to the running enrichment-score maximum for positively enriched sets or to the minimum for negatively enriched sets. Benjamini–Hochberg false discovery rate correction was applied across all 7446 eligible C2 gene sets from the joint model, rather than only the four MSI-related signatures displayed in Figure 3A–D.

The MANTIS (Microsatellite Analysis for Normal Tumor InStability) score is assessed based on NGS data, which can demonstrate samples’ MSI status. This score has a sensitivity of 100%, specificity of 97.4%, and accuracy of 98.7% for predicting MSI status in colorectal cancer patients [28]. We obtained the MSI-MANTIS scores and survival data (including overall survival data, disease-free interval data, disease-specific survival data and progression-free interval data) of TCGA-CRC patients from the cBioPortal database. For Figure 3E, individual TCGA-CRC tumors were ordered by their MSI-MANTIS scores and displayed as a bar plot, with one bar representing each tumor. Tumors were classified as MSI-H or MSS using the recommended MSI-MANTIS cutoff of 0.4 [29]. Tumors with an MSI-MANTIS score ≥ 0.4 were classified as MSI-H, whereas those with a score < 0.4 were classified as MSS, according to the recommended cutoff [28,29]. Survival analyses were performed separately by endpoint among patients with available endpoint-specific records; valid records with zero follow-up time were retained. Kaplan–Meier curves were compared using the log-rank test, and univariable Cox models were used to estimate hazard ratios (HRs) and 95% confidence intervals. Curves were displayed through 60 months, whereas inferential tests used the full available follow-up; numbers at risk were shown at 0, 12, 24, 36, and 60 months. The BH adjustment for the prespecified survival family covered OS, DSS, DFI, and PFI. Given the sparse events in the *PROKR2*-altered MSI-H subgroup, univariable Firth-penalized Cox models were additionally fitted as sensitivity analyses for OS, DSS, PFI and DFI (Supplementary Table S7). Firth-penalized HRs with profile-likelihood 95% confidence intervals and *p* values were reported.

## Figures and Tables

**Table 1:** ACMG/AMP evidence assessment and classification of the *PROKR2* and *ARHGAP35* variants identified in this family.

Evidence	*PROKR2* (NM_144773.4) c.337T>C; p.Tyr113His	*ARHGAP35* (NM_004491.4) c.3616C>G; p.Pro1206Ala
Very strong, PVS1	Not applicable	Not applicable
Strong, PS1~4	Y113H showed reduced PROK2-induced Egr1-luciferase activity and intracellular calcium mobilization, together with reduced total receptor protein expression (~53% of WT) on Western blotting [9]; Cox et al. [10] previously applied PS3 to this variant (PS3)	No variant-specific functional evidence was identified. (PS3 not applied)
	Reported in 3 unrelated Kallmann syndrome patients [9,11,12] (PS4_Supporting)	No published unrelated CHH/KS cases carrying this specific variant were identified. (PS4 not applied)
Moderate, PM1~6	The joint exome–genome frequency was 69/1,614,230 (0.00427%) overall and 61/44,886 (0.13590%) in the East Asian population. The EAS frequencies were 51/39,700 (0.12846%) in exomes and 10/5186 (0.19283%) in genomes. No homozygotes were reported in gnomAD. (PM2 not applied)	The gnomAD v4 exome frequency was 3/1,461,500 (0.000205%) overall and 3/39,698 (0.00756%) in the East Asian population. No homozygotes were reported in gnomAD. (PM2_Supporting)
Supporting, PP1~5	The variant was identified in the proband, his father, and his clinically unaffected sister. The father reported mild olfactory impairment but did not have a diagnosis of KS. These findings were insufficient to establish cosegregation with KS (PP1 not applied)	The variant was identified in the proband and his father. The father reported mild olfactory impairment but did not have a diagnosis of KS. These findings were insufficient to establish cosegregation with KS (PP1 not applied)
	Computational predictions supported a deleterious effect: SIFT, 0 (deleterious); PolyPhen-2, 1.000 (probably damaging); and AlphaMissense, 0.9317 (likely pathogenic). (PP3)	Available computational predictions did not support a deleterious effect. (PP3 not applied)
ClinVar status	Conflicting classifications of pathogenicity: likely pathogenic (1 submission), uncertain significance (3 submissions), and likely benign (1 submission). Variation ID: 338862.	No ClinVar record identified.
Result	Likely Pathogenic (1 Strong + 2 Supporting)	Variant of uncertain significance (1 Supporting)

## Data Availability

The original contributions presented in this study are included in the article/Supplementary Material. Further inquiries can be directed to the corresponding authors.

## Supplementary Materials

The following supporting information can be downloaded at: https://www.mdpi.com/article/10.3390/ijms27219703/s1.

## Abbreviations

ACMG/AMP	American College of Medical Genetics and Genomics/Association for Molecular Pathology
ADT	Androgen-deprivation therapy
BH	Benjamini–Hochberg
CHH	Congenital hypogonadotropic hypogonadism
CNV	Copy-number variation
CRC	Colorectal cancer
DFCI	Dana-Farber Cancer Institute
DFI	Disease-free interval
dMMR	Deficient mismatch repair
DSS	Disease-specific survival
EG-VEGF	Endocrine gland-derived vascular endothelial growth factor
FDR	False discovery rate
FSH	Follicle-stimulating hormone
GnRH	Gonadotropin-releasing hormone
GSEA	Gene set enrichment analysis
HCG	Human chorionic gonadotropin
HR	Hazard ratio
HRT	Hormone replacement therapy
ICI	Immune checkpoint inhibitor
IHC	Immunohistochemistry
IHH	Idiopathic hypogonadotropic hypogonadism
KS	Kallmann syndrome
LH	Luteinizing hormone
LS	Lynch syndrome
MANTIS	Microsatellite Analysis for Normal Tumor InStability
MLPA	Multiplex ligation-dependent probe amplification
MMR	Mismatch repair
MSI	Microsatellite instability
MSI-H	Microsatellite instability-high
MSS	Microsatellite stable
NES	Normalized enrichment score
NGS	Next-generation sequencing
nIHH	Normosmic idiopathic hypogonadotropic hypogonadism
OR	Odds ratio
OS	Overall survival
PFI	Progression-free interval
PROK1	Prokineticin 1
PROK2	Prokineticin 2
PROKR1	Prokineticin receptor 1
PROKR2	Prokineticin receptor 2
TCGA	The Cancer Genome Atlas
TMB	Tumor mutational burden
VUS	Variant of uncertain significance
WES	Whole-exome sequencing
WT	Wild type
